# A case report: 1-year follow-up of cerebral sparganosis mansoni with a stroke-like onset

**DOI:** 10.1186/s12883-019-1335-1

**Published:** 2019-05-29

**Authors:** Dan Xie, Min Wang, Xu Chen, Hou-zhen Tuo

**Affiliations:** 1 Department of Neurology, Beijing Friendship Hospital Affiliated to Capital Medical University, No. 95th Yongan Road, Xicheng District, Beijing, 100050 China; 2 Liver Research Center, Beijing Friendship Hospital Affiliated to Capital Medical University, Beijing, 100050 China; 3 Department of Neurosurgery, Beijing Friendship Hospital Affiliated to Capital Medical University, Beijing, 100050 China

**Keywords:** Sparganosis mansoni, Intracranial infection, Stroke, Parasitic diseases, MRI, *Spirometra*

## Abstract

**Background:**

Sparganosis mansoni is a parasitic disease caused by infection with the larvae of *Spirometra* spp. tapeworms. Its clinical manifestations and severity depend on the migration and the location of the parasites. The proportion of cerebral sparganosis in all *Spirometra mansoni* infections is 13.5% in Thailand and 12.4% in China. In the clinical setting, cerebral sparganosis is often misdiagnosed due to atypical characteristics, irregular intracranial location, and atypical epidemiology.

**Case presentation:**

The patient in the case study suffered from an acute paroxysmal attack of lateral numbness, accompanied with focal epilepsy. He was admitted to the neurology department as a stroke patient but was later diagnosed with cerebral sparganosis mansoni following lab and radiology investigations. He was fully recovered and free of *Spirometra mansoni* one year after initial consultation following several courses of oral praziquantel. The current report focuses on the diagnosis, treatment and follow up of this patient.

**Conclusions:**

A case of cerebral sparganosis mansoni with a stroke-like onsetsuggests that in the clinical diagnosis, neurologists should pay attention to brain lesions and look out for the possibility of neuroparasitic infections when dealing with patients with stroke-like onset accompanied by epilepsy. Detections of relevant antibodies in blood and cerebrospinal fluid may be necessary. The combination of the epidemiological history, clinical manifestations, detection of parasite antibody, head radiology, pathological biopsy, and identification of parasites will help us in diagnosis and differential diagnosis.

## Background

Sparganosis mansoni is a disease caused by infection with the plerocercoid or procercoid larvae (sparganum) of tapeworms of the genus *Spirometra*. The most commonly encountered species responsible for causing disease in humans in Asia is *Spirometra mansoni*, while *Spirometra mansonoides* is endemic in the Americas. In extremely rare cases *Spirometra proliferum* can be the causative agent of sparganosis in man.. Three separate hosts are required to complete the complex life cycle of *Spirometra mansoni*. Water fleas, of the genus *Cyclops*, ingest the *Spirometra* spp. eggs and are the first intermediate host. Water containing the infected water fleas is ingested by the second intermediate host such as snakes, birds andmammals. These are eaten by the definitive host, usuallyeither cats or dogs, where the develop into adult worms in the host intestines. Human can become the second intermediate host, the paratenic host following ingestion of infested water of contaminated raw or undercooked meat [1].Cerebral sparganosis is a rare complication of *Spirometra* spp. infection caused by parasites invading and living in the brain. While there have been scattered reports of cerebral sparganosis around the globe, the highest incidence is seen in Asian countries such as Korea, Japan, Thailand, and China [2, 3]. This rare neuroparasitic infection is normally displayed as paroxysmal headache, epilepsy, increased intracranial pressure, and disturbance of consciousness. The time lag from onset to diagnosis is between 6-months and 11 years, with a median of 3.7 years [1]. Most patients contracting this infection live in suburban or rural areas while only a few are from the urban areas. The patient in this case study suffered an acute paroxysmal attack of lateral numbness, accompanied with focal epilepsy. He was admitted to the neurology department of Beijing Friendship hospital as a stroke patient but was later diagnosed with cerebral sparganosis following laboratory and radiology investigations. He was fully recovered and free of *Spirometra mansoni* following 1 year of interval oral praziquantel administration of. The current report focuses on the diagnosis, treatment and follow up of this patient.

## Case presentation

### Clinical summary

A 45 years old male patient, attended the neurology emergency department on 3rd January 2016 due to right limb numbness for 6 days and convulsive seizure for 3 days. Six days before the hospital visit, the patient experienced numbness in the right upper limb and instability while holding without obvious incentive but did not seek immediate medical intervention. Three days before admission, the patient experienced convulsive seizure in the right upper limb while remaining conscious, which was relieved after 1 min. Similar attacks occurred intermittently on six further occasions. The patient had a 3-year history of hypertension with the highest blood pressure being 180/110 mmHg. He also had a history of smoking and drinking lasting more than 30 years. He was born and has always lived in Beijing, with no history of contact with infested water, infectious zone, other radioactive substances or toxins. Upon admission, the patient was examined to be obese with no subcutaneous nodules. Neurological examinations showed full level muscle strength in the right upper limb, accompanied with diminished needling response. Emergency head CT scan (2016-1-1) showed lower density in the left parietal lobe. As the patient manifested as an acute onset of right limb weakness and hemiparesis,with low density lesions in the left occipital lobe on CT and a history of hypertension, the patient was hospitalised with a preliminary diagnosis of acute stroke and secondary epilepsy.

After hospitalisation, head MRI scan (2016-1-4) displayed a lesion in the left parietal lobe of unknown nature. After enhancement in the magnetic field, a larger area of oedema was found around the lesion in the left parietal lobe which could indicate glioma or other inflammatory diseases. Since the nature of the brain lesion did not match the characteristics of common cerebrovascular diseases, intracranial angiography DSA was used but found no obvious vascular abnormalities or stenosis. Further examinations including lumbar puncture, immune rheumatoid factors and parasite antibody detections were carried out. A raised cerebrospinal fluid pressure was found to (215 mm H_2_O) with no red or white blood cells present. After consultation within the neurology department, intracranial tumor was considered and therefore prepared for stereotactic biopsy of the brain. At this point, pathology results came back positive for *Spirometra mansoni* IgG. On further questioning the patient admitted that he had drank tap water and eaten frogs when travelling in another province during June–September 2015. Given his medical history, and results from head MRI and blood tests, the patient was considered to be infected with *Spirometra mansoni* and surgical intervention or antihelmintic chemotherapy was recommended .

The patient accepted pharmaceutical treatment and was given praziquantel (1600 mg, 20 mg/kg) 3 times a day for 10 days. During these 10 days, the patient reported occasional headache and was treated for dehydration before discharge from the hospital. The patient was also administration oral sodium valproate 500 mg 3 times a day to control seizures.

The patient was hospitalised again in March and July 2016 and treated with praziquantel (1600 mg,20 mg/kg) 3 times a day for 10 days. His headaches were eased with intravenous infusion of 20% 250 ml mannitol twice a day.

Lumbar puncture (Table 1 Examination of cerebrospinal fluid), head MRI, blood biochemistry, conventional blood analysis and parasite antibody examinations (Table 2 Spirometra mansoni IgG antibody) were also carried out on both occasions. On 13th Jul 2016, the patient was free from numbness and seizures in the upper limb.

**Table 1:** Examination of cerebrospinal fluid

	2016/01/11	2016/01/21	2016/03/24	2016/07/06
Coagulation	No	No	No	No
Color	Clear	Clear	Clear	Clear
Aspect	Clear	Clear	Cloudy	Clear
Pandy’s test	Negative	Negative	Negative	Negative
White blood cells (× 10^6^/L)	0	0	5	0
Red blood cells (×10^6^/L)	0	10	30	0
Protein (mg/dl)	22.9	29.91	24.43	24.32
Potassium (mmol/L)	2.78	2.95	2.81	2.76
Sodium (mmol/L)	149.1	151.4	148.3	149.8
Chloride (mmol/L)	121.8	124.8	120.4	120.6
Glucose (mmol/L)	3.42	3.92	3.14	3.56
Calcium (mmol/L)	1.2	1.1	1.2	1.2

**Table 2:** Spirometra mansoni IgG antibody

2016/01/14	2016/03/30	2016/07/13
Positive	Positive	Negative

#### Pathological findings

##### Laboratory results

Table 1 displays the laboratory results of lumbar puncture performed on the three occasions when he was admitted to hospital and during the 1-year follow-up appointment. CSF analysis showed normal results except for a few white and red blood cells during his second hospital stay most likely resulting from the procedure itself. Table 2 shows the *Spirometra mansoni* IgG result on three occasions. Note that the IgG result became negative during his third hospital stay following three courses of praziquantel treatment.,.

##### Radiology results

**Fig. 1:**
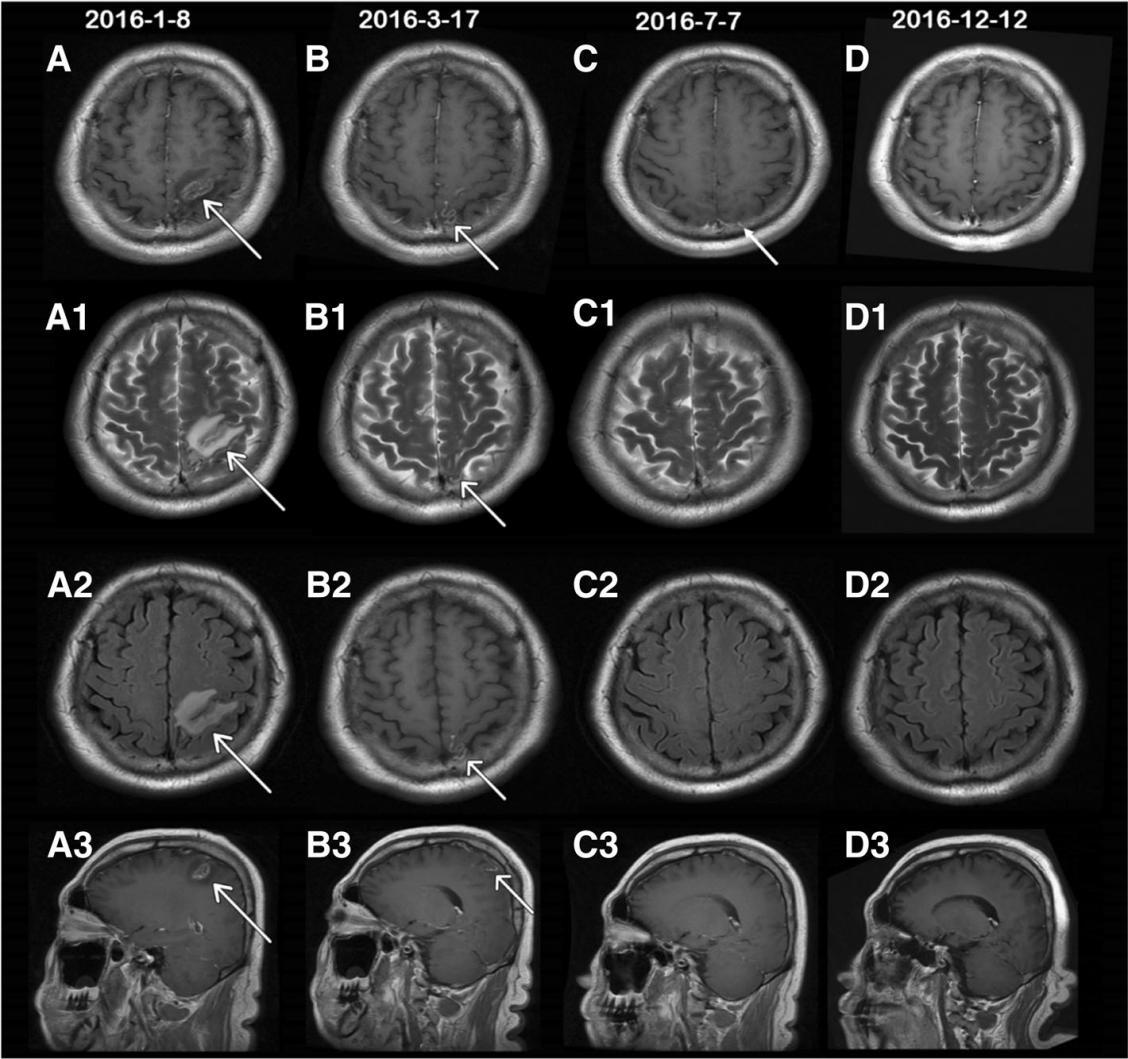
head MRI scan during three hospital stays and 1 year follow-up. **a**-**d** T1WI + FLAIR+contrast enhancement MRI on 4 occasions; A1-D1 T2WI; A2-D2 T2WI + FLAIR; A3-D3 sagittal T1WI + FLAIR+contrast enhancement. A. A3 shows MRI scan during the first hospital stay in January 2016. T1WI with arrowhead shows horseshoe lesion with short signal and irregular enhancement. Both T2WI and T2WI + FLAIR have long signals. B.B3 shows MRI scan in March 2016 showing veil-like long T1 and long T2 signals in the parietal lobe with obvious enhancement. This is considered to be the dead parasite bodies after deworming treatment. Tunnel sign caused by parasite migration is shown in B3 with arrow. C.C3 shows MRI scan in July 2016. The arrow indicates few spot enhancements in T1 + C with minimum lesion present. D.D4 shows MRI scan in the first-year follow-up in December 2016. No obvious abnormal lesions were seen

Enhanced head MRI scans were performed during the three hospital stays in January, March, and July 2016. Figure 1 A-A3display head MRI scans performed on 8th January. The scans showedan abnormal horseshoe signal in the left parietal lobe with a low T1WI signal and a high T2WI + FLAIR signal. Enhanced scan showed irregular wreath in the lesion without enhancement in the surrounding. This type of abnormality in the left parietal lobe may indicate glioma. During the second hospital stay, the MRI scan carried out on 17th March showed abnormal small stripes of signal shadow in the left parietal lobe with a decreased range. It also showed clearer abnormal veil-like signal shadow in the left parietal lobe as compared to the previous MRI scan. These results may indicatethe presence of *Spirometra* within this area of the brain. During the third hospital stay, MRI scan on 7th July detected only minor abnormalities in the bilateral frontal lobes and parietal lobes indicating that the lesions has reduced significantly or resolved. The follow-up on 12th December showed no abnormality in the head MRI scan. MRI scans from each hospital visit are shown in Fig. 1.

## Discussion and conclusions

With the improvement of people’s living standards, changes in eating habits, and diversities in dietary sources and styles, the incidence of foodborne parasitic diseases hasgradually increased [4]. Some neuroparasitic diseases are foodborne, with close association with the consumption of raw or undercooked fish, prawns, meat and frogs. Wang et al. [5] studied 24 cases of Sparganosis mansoni and found that consumptions of raw or undercooked snakes, frogs, tadpoles or infested water accounted for 79.2% of all cases.. The source of the infection was unclear in the remaining 20.8% of cases. However, the patient in the current report only admitted having previously consumed potentially infested water when the *Spirometra mansoni* antibody test returned a positive result. Therefore, in the process of clinical diagnosis, acknowledgement of epidemiological history is required for a comprehensive analysis and diagnosis.

The symptoms of Sparganosis mansoni are complex and diverse including various clinical manifestations, irregular location of the parasite in the brain and untypical epidemiological history. Patients normally present with focal neurological dysfunction including chronic paroxysmal headache, epilepsy, increased intracranial pressure, disturbance of consciousness, limb numbness and visual impairment. Epilepsy being the most commonly encountered symptom, present in 45.8 to 51.8% patients who develop cerebral sparganosis [5, 6]. Due to the lack of specific clinical manifestations indicating *Spirometra* infection, this disease normally has a chronic course with an extremely high rate of misdiagnosis. All 24 patients in Wang’s study were initially misdiagnosed with conditions including glioma, brain abscess, brain tuberculosis and primary epilepsy with the longest time of misdiagnosis of 11 years [5]. In a study of 27 neuroparasitic infections, Chen et al. [6] reported a 74.4% misdiagnosis rate. The patient in the current study presented with an acute stroke-like onset with a history of hypertension, drinking and smoking; and was therefore admitted as a stroke patient. However, the presence of focal seizures during the onset, the irregularity in the vasculature shown by MRI, the absence of stenosis in intracranial angiography, and the obvious enhancement in enhanced MRI scan indicated the possibility of inflammation and tumour. Sparganosis was diagnosed only following the detection of *Spirometra mansoni* antibody. This case report demonstrates the importance of acquiring an accurate medical history, and performing a lumbar puncture and parasitic antibody testing to rule out the possibility of atypical neuroparasitic disease when the patient with acute stroke-like symptoms with seizures, is in the high-risk group of cerebrovascular disease, with multiple or no obvious vascular distribution in the lesion or when the lesion shape does not meet with the vasculature.

At the moment, the diagnosis of Sparganosis mansoni is mainly based on clinical symptoms, laboratory testing of blood and cerebrospinal fluid, head MRI and surgical pathology. Laboratory tests mainly rely on the demonstration of a raised white blood cell count, an increase in eosinophils, an increased CD4/CD8 ratio, anddetection of *Spirometra mansoni* detection using ELISA. An increase in white blood cell count and protein concentration in the cerebrospinal fluid may also be present. The most specific and accurate test is the detection of the parasite antibody. In the 24 cases screened by Wang et al. [5], 22/24 (91.7%) cases reported positive detection of *Spirometra mansoni* IgG antibody. In the current case study, the patient had no abnormalities in the blood or cerebrospinal fluid and therefore the diagnosis was mainly based on the detection of the parasite antibody. The presence of parasite antibody persisted during the subsequent hospital stays and the duration was parallel to the disappearance of intracranial lesions.

Head MRI scan is an effective aid when diagnosing Sparganosis mansoni. In 1993, Moon et al. [7] first reported the MRI scan in Sparganosis, showing white matter degeneration, as indicated by weak T1WI signal and strong T2WI signal in the low density CT. Gong et al. [8] reported that the rate of accurate diagnosis of cerebral Sparganosis mansoni could be increased from 0 to 11.8% and 28.6% with multiple follow-up examinations. Song et al. [9] suggested that the most typical MRI manifestation was tunnel-like or rope-like, with the most common being bead-shaped enhancement. In the current case, the first enhanced MRI scan showed horseshoe-like lesions, with lace-like enhancement without obvious characteristics. However, the subsequent MRI scan in March 2016 showed an obvious tunnel-like signal with enhanced parasite body. is the caase reported here agrees with the finding of both the Gong and Song studies and highlight the importance of performing multiple enhance head MRI scans in the diagnosis of Sparganosis mansoni. Pathological examination of the excised parasite was not completed in the current study to confirm diagnosis due to the refusal of surgical intervention by the patient. However, the clinical symptoms,CNS lesions and the presence of *Spirometra mansoni* antibodies had disappeared by the 1 year follow up following anthelmintic treatment indicating the diagnosis of sparganosis was most likely correct..

In conclusion, variability in the clinical manifestations together with the presence of complex cerebral lesions lead to high rates of misdiagnosis, resulting in delayed or missed diagnosis in patients with Sparganosis. In the current case, the patient was in a high-risk group for cerebrovascular disease with and presented with a stroke-like onset, accompanied by epilepsy-like seizures and the presence of abnormal lesions in MRI scans. The correct diagnosis was only made after detection of parasite antibody followed by Praziquantel drug treatment. During the one year follow up, dynamic changes in the brain lesions and reduction in *Spirometra mansoni* IgG antibody were discovered, and the two were mostly parallel to each other. This case suggests that in the clinical diagnosis, neurologists should carefully assess brain lesions and consider the possibility of neuroparasitic infections when dealing with patients with stroke-like onset accompanied by epilepsy. A combination of epidemiological history, clinical manifestations, detection of parasite antibody, head radiology, pathological biopsy, and identification of parasites is required to guide diagnosis diagnosis and differential diagnosis in this group of patients.

## Abbreviations

CNS: Central Nervous System
CT: Computed Tomography
DSA: Digital Subtraction Angiography
ELISA: Enzyme-Linked Immunosorbent Assay
MRI: Magnetic Resonance Imaging
